# Supplementary material for: Immunomodulatory fecal metabolites are associated with mortality in COVID-19 patients with respiratory failure
Source: Nat Commun. 2022 Nov 3;13:6615. doi: 10.1038/s41467-022-34260-2 (PMC9633022; doi:10.1038/s41467-022-34260-2)
Supplement: Supplementary file 1 — Supplementary Information [file 41467_2022_34260_MOESM1_ESM.pdf]

**Supplementary Table 1: Patient characteristics Stratified by Trajectory of Respiratory Failure**

| | HFNC Success | HFNC Failure | p |
|---|---|---|---|
| n | 30 | 20 | |
| **Baseline Characteristics** | | | |
| Race (%) | | | 0.543 |
| Asian/Mideast Indian | 1 ( 3.3) | 0 ( 0.0) | |
| Black/African-American | 20 (66.7) | 13 (65.0) | |
| Hispanic | 1 ( 3.3) | 3 (15.0) | |
| More than one Race | 1 ( 3.3) | 0 ( 0.0) | |
| Native Hawaiian/Pacific Islander | 1 ( 3.3) | 0 ( 0.0) | |
| White | 6 (20.0) | 4 (20.0) | |
| Male (%) | 15 (50.0) | 12 (60.0) | 0.685 |
| Age (median [IQR]) | 59.47 [54.31, 67.43] | 67.96 [59.28, 76.10] | 0.113 |
| Body Mass Index (median [IQR]) | 31.05 [27.30, 38.80] | 30.73 [26.95, 33.77] | 0.759 |
| Hypertension (%) | 20 (66.7) | 12 (60.0) | 0.857 |
| Hyperlipidemia (%) | 8 (26.7) | 9 (45.0) | 0.3 |
| Diabetes (%) | 11 (36.7) | 9 (45.0) | 0.768 |
| Cancer (%) | 6 (20.0) | 3 (15.0) | 0.94 |
| Chronic Kidney Disease (%) | 1 ( 3.3) | 4 (20.0) | 0.149 |
| | | | |
| **Clinical Characteristics** | | | |
| SOFA Score (median [IQR]) | 4.00 [4.00, 5.75] | 9.00 [8.75, 9.00] | 0.001 |
| APACHE Score (median [IQR]) | 18.00 [13.00, 21.25] | 21.00 [16.75, 29.50] | 0.033 |
| Charlson Comorbidity Index (median [IQR]) | 3.00 [2.00, 4.75] | 4.00 [2.00, 6.00] | 0.361 |
| Days from Symptom Onset (median [IQR]) | 5.00 [3.00, 6.75] | 5.00 [2.00, 8.00] | 0.905 |
| Admission (%) | | | 0.495 |
| Emergency Department | 22 (73.3) | 12 (60.0) | |
| Hospital Medicine | 6 (20.0) | 7 (35.0) | |
| Outside Hospital | 2 ( 6.7) | 1 ( 5.0) | |
| | | | |
| **Medication Administration** | | | |
| Antivirals (%) | | | |
| Steroid Treatment | 22 (73.3) | 18 (90.0) | 0.279 |
| Remdesivir Treatment | 24 (80.0) | 18 (90.0) | 0.581 |
| Antibiotics (%) | | | |
| Betalactams | 5 (16.7) | 4 (20.0) | 1 |
| Levofloxicin | 1 ( 3.3) | 0 ( 0.0) | 1 |
| Vancomycin | 6 (20.0) | 6 (30.0) | 0.636 |
| Metronidazole | 1 ( 3.3) | 1 ( 5.0) | 1 |
| Macrolides | 7 (23.3) | 7 (35.0) | 0.563 |
| Doxycycline | 4 (13.3) | 0 ( 0.0) | 0.242 |
| Trimethoprim-Sulfamethoxazole | 4 (13.3) | 2 (10.0) | 1 |

Supplementary Table 1: Univariable analysis comparing patient characteristics and medication administration for high flow nasal cannula (HFNC) failure, defined as need for endotracheal intubation versus HFNC success, defined as transition to low flow nasal cannula. Categorical variables absolute number (percent within group). Categorical variables were compared using a two-tailed, chi-squared test, while continuous variables were compared using the Wilcoxon rank-sum, two-tailed test (n = 71 independent samples from patients). Unadjusted p-values are presented as exact values. Adequate treatment with COVID-19 specific therapy included at least three consecutive days of therapy during index hospitalization. Sufficient total dose of dexamethasone was 18mg and Remdesivir was 400mg. Only antibiotics received 72 hours prior to fecal specimen collection are represented.

## Supplementary Table 2: Assay 1 Analyzed Compounds – PFBBr Derivatization

| Common name | Mass to Charge *m/z* $[M\text{-}H]^{-}$ or [M+PFB-H]- | Retention time (min) |
|---|---|---|
| 2-hydroxy-3-methylbutyrate | 117 | 6.39 |
| 2-hydroxyhexanoate | 131 | 7.03 |
| 2-hydroxyisocaproate | 131 | 6.80 |
| 2-methylbutyrate | 101 | 5.69 |
| 3-aminoisobutyrate | 282 | 8.85 |
| 4-methylvalerate | 115 | 6.31 |
| 5-aminovalerate | 476 | 11.07 |
| acetate | 59 | 4.44 |
| alanine | 268 | 8.36 |
| aspartate | 492 | 11.10 |
| benzoate | 121 | 7.53 |
| butyrate | 87 | 5.48 |
| catechol | 269 | 9.25 |
| crotonate | 85 | 5.79 |
| cysteine | 480 | 11.21 |
| desaminotyrosine | 345 | 11.02 |
| dopamine | 512 | 12.81 |
| glutamate | 506 | 11.48 |
| glycine | 434 | 10.05 |
| hexanoate | 115 | 6.49 |
| histamine | 430 | 11.76 |
| hydrocinnamate | 149 | 8.20 |
| indole-3-acetate | 174 | 10.19 |
| indole-3-carboxaldehyde | 144 | 9.95 |
| indole-3-propionate | 188 | 10.52 |
| isobutyrate | 87 | 5.20 |
| isoleucine | 310 | 8.97 |
| isovaleric acid | 101 | 5.74 |
| leucine | 310 | 8.91 |
| lysine | 505 | 12.01 |
| methionine | 328 | 9.97 |
| palmitate | 255 | 10.51 |
| p-cresol | 107 | 7.09 |
| phenol | 93 | 6.61 |
| phenylacetate | 135 | 7.69 |
| phenylalanine | 344 | 10.37 |
| proline | 294 | 9.19 |
| propionate | 73 | 4.98 |
| p-toluate | 135 | 8.076 |

| | | |
|---|---|---|
| serine | 464 | 10.69 |
| succinate | 297 | 9.26 |
| synephrine | 346 | 11.27 |
| threonine | 478 | 10.52 |
| trans-indole-3-acrylate | 186 | 11.93 |
| tryptamine | 500 | 11.69 |
| tryptophan | 383 | 12.74 |
| tyramine | 316 | 10.71 |
| tyrosine | 373 | 11.42 |
| valerate | 101 | 6.00 |
| valine | 296 | 8.67 |

## Supplementary Table 3: Assay 2 Analyzed Compounds – Bile Acids

| Formal Name | Molecular Formula | Neutral Mass | Common name | Mass to Charge *m/z* $[M-H]^-$ | Retention Time (+/- 0.15 min within run, +/- 0.40 min across batches) |
|---|---|---|---|---|---|
| 5β-Cholanic acid-3α, 7α, 12α-triol | C24H40O5 | 408.2876 | Cholic acid | 407.2798 | 11.75 |
| 5β-Cholanic acid-3α, 12α-diol | C24H40O4 | 392.2927 | Deoxycholic acid | 391.2849 | 15.27 |
| 5β-Cholanic acid-3α-ol | C24H40O3 | 376.2977 | Lithocholic acid | 375.2899 | 18.06 |
| 5β-Cholanic acid-3α, 7α, 12α-triol N-(carboxymethyl)-amide | C26H43NO6 | 465.309 | Glycocholic acid | 464.3012 | 8.73 |
| 5β-Cholanic acid-3α, 7α, 12α-triol N-(2-sulphoethyl)-amide | C26H45NO7S | 515.2917 | Taurocholic acid | 514.2839 | 4.97 |
| 5β-Cholanic acid-3β, 12α-diol | C24H40O4 | 392.2927 | Isodeoxycholic acid | 391.2849 | 13.24 |
| 5α-Cholanic acid-3β-ol | C24H40O3 | 376.2978 | Alloisolithocholic acid | 375.2900 | 16.46 |
| 3-Oxo-5β-cholanoic acid | C24H38O3 | 374.2821 | 3-oxolithocholic acid | 373.2743 | 18.62 |
| 5β-Cholanic acid-3α, 6β, 7α-triol-N-(2-sulphoethyl)-amide | C26H45NO7S | 515.2917 | Tauro-α-muricholic acid | 514.2839 | 2.35 |
| 5ß-Cholanic acid-3α,6ß,7ß-triol N-(2-sulphoethyl)-amide | C26H45NO7S | 515.2917 | Tauro-β-muricholic acid | 514.2839 | 2.35 |
| 5β-Cholanic acid-3, 7, 12-trione-N-(carboxymethyl)-amide | C26H37NO6 | 459.2621 | Glycodehydrocholic acid | 458.2543 | 1.90 |
| 5β-Cholanic acid-3α, 7α-diol N-(carboxymethyl)-amide | C26H43NO5 | 449.3141 | Glycochenodeoxycho-lic acid | 448.3063 | 11.97 |
| 5β-Cholanic acid-3α, 12α-diol N-(carboxymethyl)-amide | C26H43NO5 | 449.3141 | Glycodeoxycholic acid | 448.3063 | 12.56 |
| 5β-Cholanic acid-3α, 7β-diol N-(carboxymethyl)-amide | C26H43NO5 | 449.3141 | Glycoursodeoxycholic acid | 448.3063 | 8.26 |
| 5β-Cholanic acid-3α-ol N-(carboxymethyl)-amide | C26H43NO4 | 433.3192 | Glycolithocholic acid | 432.3114 | 15.23 |

| | | | | | |
|---|---|---|---|---|---|
| 5ß-Cholanic acid-3α,6α-diol N-(carboxymethyl)-amide | C26H43NO5 | 449.3100 | Glycohyodeoxycholic acid | 448.3022 | 8.70 |
| 5β-Cholanic acid-3α, 6α, 7β-triol | C24H40O5 | 408.2876 | ω-muricholic acid | 407.2798 | 9.07 |
| 5β-Cholanic acid-3α, 6β, 7β-triol | C24H40O5 | 408.2876 | β-Muricholic acid | 407.2798 | 10.22 |
| 5β-Cholanic acid-3α, 6α, 7α-triol | C24H40O5 | 408.2876 | γ-Muricholic acid | 407.2798 | 11.10 |
| 5β-Cholanic acid-3α, 6β, 7α-triol | C24H40O5 | 408.2876 | α-Muricholic acid | 407.2798 | 9.90 |
| 3α,7α,12α-Trihydroxy-5α-cholan-24-oic acid | C24H40O5 | 408.2876 | Allocholic acid | 407.2798 | 11.62 |
| 5β-Cholanic acid-3α,7α-diol | C24H40O4 | 392.2927 | Chenodeoxycholic acid | 391.2849 | 14.87 |
| 5α-Cholanic acid-3α-ol | C24H40O3 | 376.2978 | Allolithocholic acid | 375.2900 | 18.20 |
| 5β-Cholanic acid-3α, 7β-diol | C24H40O4 | 392.2927 | Ursodeoxycholic acid | 391.2849 | 11.88 |
| 3α,6α-Dihydroxy-5β-cholan-24-oic acid | C24H40O4 | 392.2927 | Hyodeoxycholic acid | 391.2849 | 12.23 |
| 5β-Cholanic acid-7α, 12α-diol | C24H40O4 | 392.2927 | 3-Deoxycholic acid | 391.2849 | 17.35 |
| 5β-Cholanic acid-3β-ol | C24H40O3 | 376.2977 | Isolithocholic acid | 375.2899 | 17.01 |
| 5β-Cholanic acid-3α, 7α-diol-12-one | C24H38O5 | 406.2719 | 12-Oxochenodeoxycholic acid | 405.2641 | 8.61 |
| 5β-Cholanic acid-3α, 12α-diol-7-one | C24H38O5 | 406.2719 | 7-Oxodeoxycholic acid | 405.2641 | 8.96 |
| 5β-Cholanic acid-7α,12α-diol-3-one | C24H38O5 | 406.2719 | 3-Oxocholic acid | 405.2641 | 10.70 |
| 5β-Cholanic acid-12α-ol-3-one | C24H38O4 | 390.277 | 3-Oxodeoxycholic acid | 389.2692 | 14.76 |
| 5β-Cholanic acid-7α-ol-3-one | C24H38O4 | 390.277 | 3-Oxochenodeoxycholic acid | 389.2692 | 14.76 |
| 5β-Cholanic acid-3α-ol-7-one | C24H38O4 | 390.277 | 7-Oxolithocholic acid | 389.2692 | 12.37 |
| 5β-Cholanic acid-3α-ol-6-one | C24H38O4 | 390.277 | 6-Oxolithocholic acid | 389.2692 | 12.37 |
| 5β-Cholanic acid-3α-ol-12-one | C24H38O4 | 390.277 | 12-Oxolithocholic acid | 389.2692 | 12.67 |
| 5β-Cholanic acid-3α, 12α-diol N-(2-sulphoethyl)-amide | C26H45NO6S | 499.2968 | Taurodeoxycholic acid | 498.289 | 9.35 |
| 5β-Cholanic acid-3α-ol N-(2-sulphoethyl)-amide | C26H45NO5S | 483.3018 | Taurolithocholic acid | 482.294 | 11.80 |
| 5β-Cholanic acid-3α, 7α-diol N-(2-sulphoethyl)-amide | C26H45NO6S | 499.2968 | Taurochenodeoxycholic acid | 498.289 | 8.70 |

| | | | | | |
|---|---|---|---|---|---|
| 3α,7β-Dihydroxy-5β-cholan-24-oic acid N-(2-sulfoethyl)amide | $C_{26}H_{45}NO_6S$ | 499.2968 | Tauroursodeoxycholic acid | 498.289 | 4.30 |
| 5β-Cholanic acid-3α, 6α-diol-N-(2-sulphoethyl)-amide | $C_{26}H_{45}NO_6S$ | 499.2968 | Taurohyodeoxycholic acid | 498.289 | 4.60 |
| 5β-Cholanic acid-3α-ol-7,12-dione | $C_{24}H_{36}O_5$ | 404.2563 | 3-Hydroxy-7,12-diketolithocholic acid | 403.2485 | 5.00 |
| 3α,7β,12α-Trihydroxy-5β-cholan-24-oic acid | $C_{24}H_{40}O_5$ | 408.2876 | Ursocholic acid | 407.2798 | 6.75 |
| D4-Cholic acid | $C_{24}H_{36}D_4O_5$ | 412.3126 | D4-Cholic acid | 411.3048 | 11.76 |
| D4-Deoxycholic acid | $C_{24}H_{36}D_4O_4$ | 396.3177 | D4-Deoxycholic acid | 395.3099 | 15.20 |
| D4-Glycocholic acid | $C_{26}H_{39}D_4NO_6$ | 469.3341 | D4-Glycocholic acid | 468.3263 | 8.73 |
| D4-Glycodeoxycholic acid | $C_{26}H_{39}D_4NO_5$ | 453.3392 | D4-Glycodeoxycholic acid | 452.3314 | 12.50 |
| D4-Lithocholic acid | $C_{24}H_{36}D_4O_3$ | 380.3228 | D4-Litchocholic acid | 379.3150 | 18.00 |
| D4-Taurocholic acid | $C_{26}H_{41}D_4NO_7S$ | 519.3167 | D4-Taurocholic acid | 518.3089 | 5.01 |
| D4-Taurodeoxycholic acid | $C_{26}H_{41}D_4NO_6S$ | 503.3218 | D4-Taurodeoxycholic acid | 502.3140 | 9.40 |
| D5-Alpha-muricholic acid | $C_{24}H_{35}D_5O_5$ | 413.3189 | D5-Alpha-muricholic acid | 412.3111 | 9.85 |
| D4-Taurochenodeoxycholic acid | $C_{26}H_{41}D_4NO_6S$ | 503.3218 | D4-Taurochenodeoxycholic acid | 502.3140 | 8.70 |

**Supplementary Figure 1: Consort Diagram**

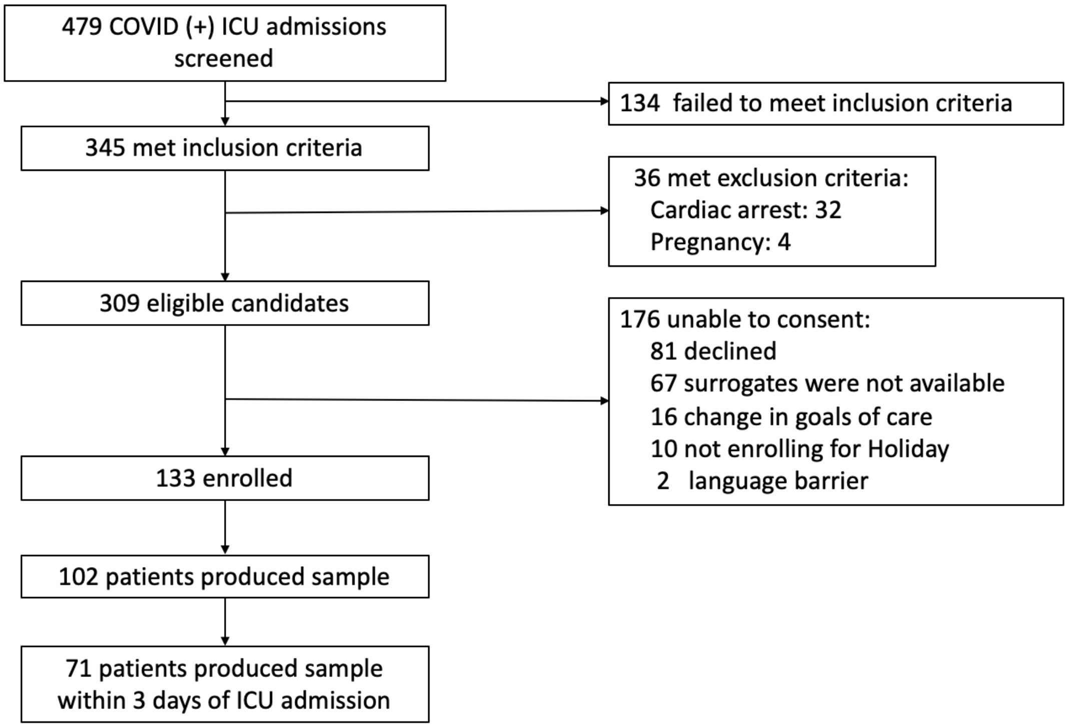


Supplementary Figure 1 Legend: Consort diagram for study enrollment.

## Supplementary Figure 2

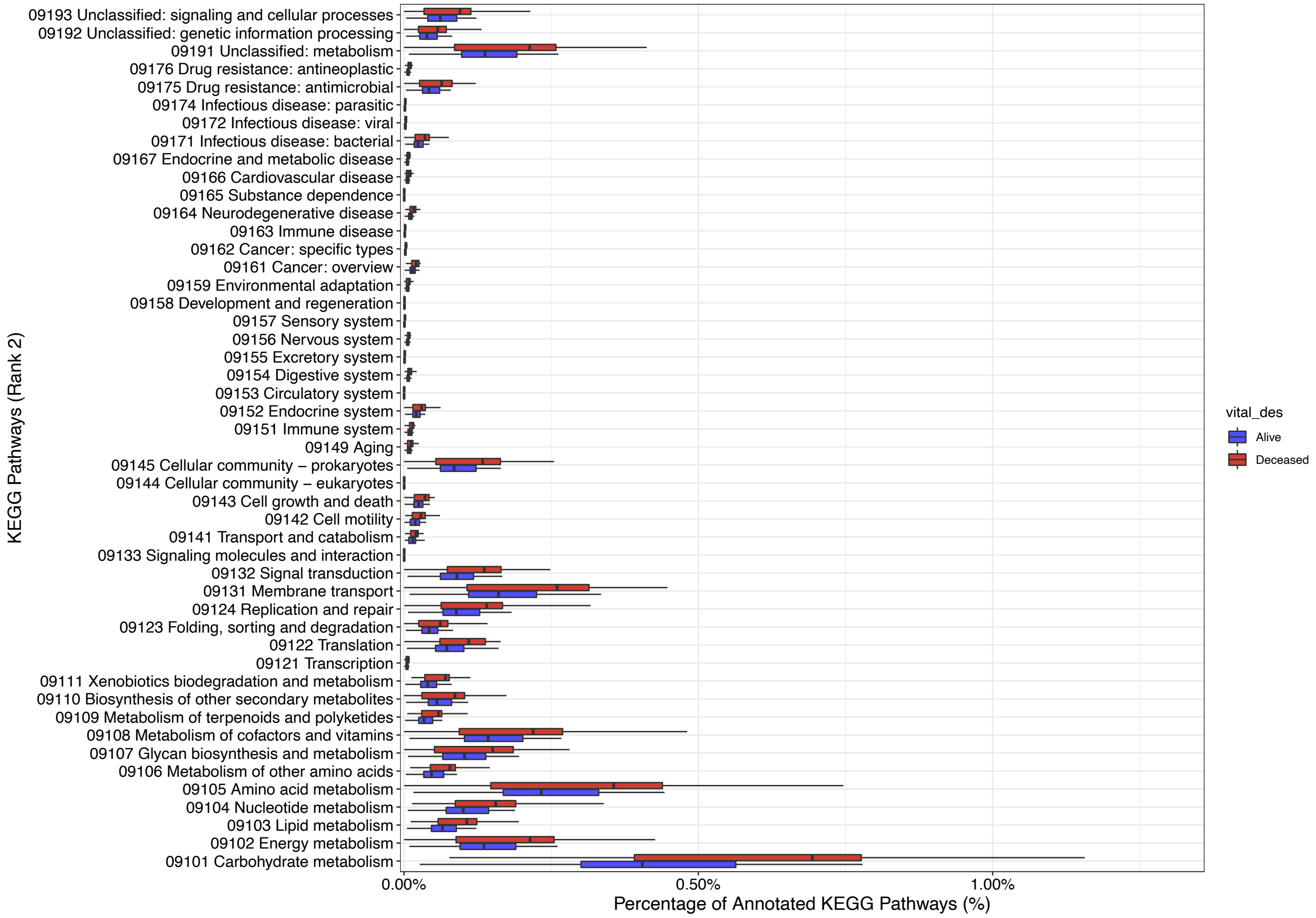


Supplementary Figure 2 Legend: KEGG pathway analysis stratified by survival outcome (blue box: alive and red box: deceased). KEGG pathways (Rank 2) were calculated as a total percent of all pathways encoded and plotted as boxplots with values per each rank 2 category. After performing a two-tailed, Wilcoxon rank-sum test, there were no statistical differences across all KEGG pathways shown (n = 71 independent samples from patients). Boxes show interquartile ranges (IQR) where the center black line represents the median and the whiskers (vertical black lines) extend to 1.5 x IQR or to the minimum and maximum value, whichever is closest to the median

## Supplementary Figure 3

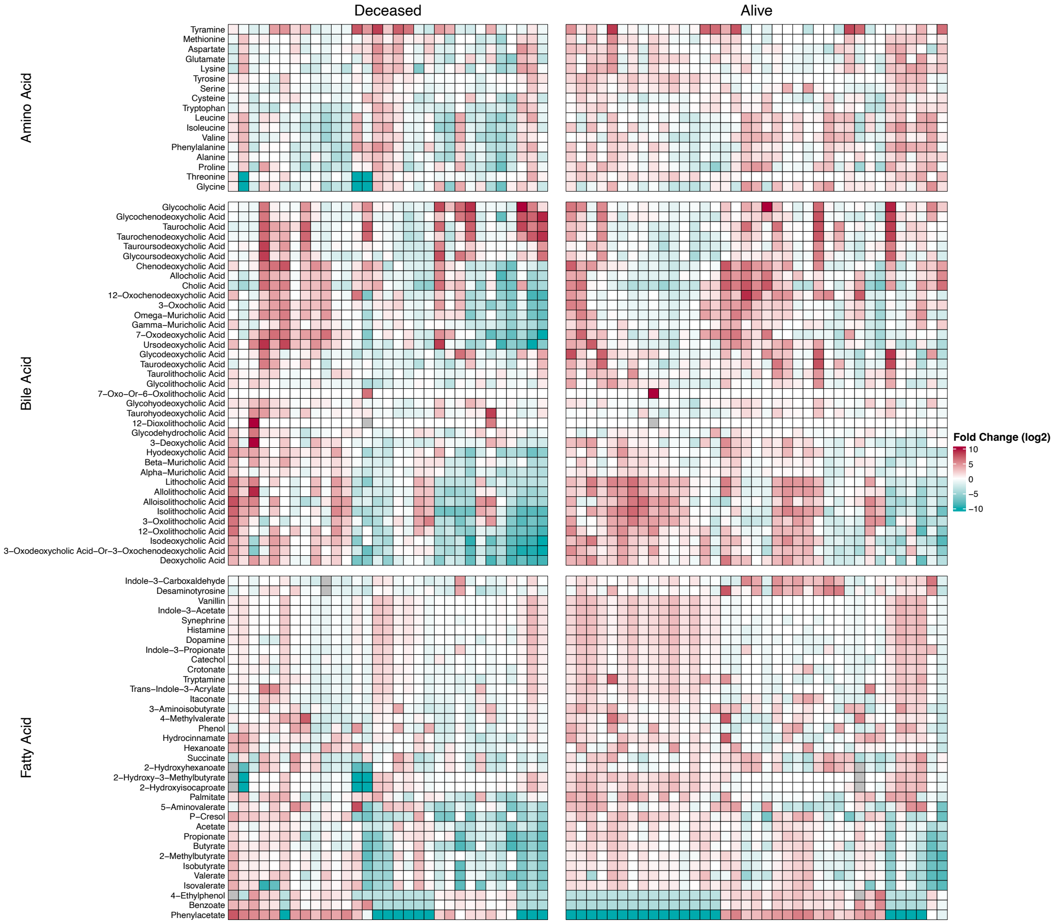


Supplementary Figure 3 Legend: Heatmap of 89 relatively quantified fecal metabolites. Dark cyan colors correlate to low log2 fold-change values relative to the median value per compound, while dark red colors correlate to high log2 fold-change values relative to the median value per compound. Missing values are shown in gray. n = 71 independent samples from patients.

## Supplementary Methods:

### Supplementary Metabolomic Methods (MetaboLights study MTBLS5288)

### Stool Collection and Extraction for All Metabolomic Assays

***Collection:*** Freshly obtained stool samples were collected from patients in a sterile specimen vessel (University of Chicago, IRB 20-1102). Samples were immediately placed on ice or at 4 °C and then immediately aliquoted for downstream analyses. ***Stool Splitting and Storage:*** Stool samples were then split into aliquots for downstream analyses (metabolomics, genomics). For each analysis, stool was split into a separate 1.5 ml Eppendorf tube. Mass requirements for analyses were: metabolomics, ~100 mg; genomics, ~250 mg (16S and shotgun sequencing). Minimum requirements for metabolomic analyses in this study was 20 mg/assay. The bulk stool sample and split Eppendorf tubes were kept on ice and the aliquots were immediately stored at -80 °C. ***Extraction:*** Extraction solvent (80% methanol spiked with internal standards and stored at -80 °C) was added to pre-weighed fecal samples at a ratio of 100 mg of material/ml of extraction solvent in beadruptor tubes (Fisherbrand; 15-340-154). Samples were homogenized at 4 °C on a Bead Mill 24 Homogenizer (Fisher; 15-340-163), set at 1.6 m/s with 6 x 30s cycles, 5 s off per cycle. Samples were then centrifuged at -10 °C, 20,000 x g for 15 min and the supernatant was used for subsequent metabolomic analysis

### Assay 1 (CI-GC-MS): Pentafluorobenzyl bromide (PFBBr) derivatization for targeted metabolomics with (-)CI-GCMS (quantitative and normalized relative abundance)

***Preparation for Pentafluorobenzyl Bromide (PFBBr) Derivatization:*** Samples were derivatized as previously described with the following modifications [1]. Briefly, the metabolite extract (100 µl) was added to 100 µl of 100 mM borate buffer (pH 10) (Thermo Fisher, 28341), 400 µl of 100 mM pentafluorobenzyl bromide (Millipore Sigma; 90257) in acetonitrile (Fisher; A955-4), and 400 µl of n-hexane (Acros Organics; 160780010) in a capped mass spectrometer autosampler vial (Microliter; 09-1200). Samples were heated in a thermomixer C (Eppendorf) to 65 °C for 1 h while shaking at 1300 rpm. After cooling to RT, samples were centrifuged at 4 °C, 2000 x g for 5 min, allowing phase separation. The hexanes phase (100 µl) (top layer) was transferred to an autosampler vial containing a glass insert and the vial was sealed. Another 100 µl of the hexanes phase was diluted with 900 µl of n-hexane in an autosampler vial. ***Chromatography:*** Concentrated and dilute samples were analyzed using a GC-MS (Agilent 8890/7890B GC system, Agilent 5977B MSD) with a 1 µl split injection (1:10 split ratio). Oven ramp parameters: 1 min hold at 60 °C, 25 °C/min up to 300 °C with a 2.5 min hold at 300 °C. Inlet temperature was 280 °C and transfer line was 310 °C. Using a HP-5ms ultra inert column (30 m x 0.25 mm, 0.25 µm; Agilent Technologies 19091S-433UI), methane as the reagent gas. ***Instrument Settings:*** The instrument was in Scan acquisition mode (m/z 50-600) with a solvent delay of 4.2 with Normal Scanning and operating in negative chemical ionization mode with methane used as the reagent gas (99.999% pure). ***Data Transformation:*** A 10-point calibration curve was prepared with acetate (100 mM), propionate (25 mM), butyrate (12.5 mM), and succinate (50 mM), with 9 subsequent 2x serial dilutions. Data analysis was performed using **MassHunter Quantitative Analysis software (version B.10, Agilent Technologies)** and confirmed by comparison to retention time, nominal mass, and fragmentation patterns of authentic standards (**Supplementary Table 2**). Normalized peak areas were calculated by dividing raw peak areas of targeted analytes by raw peak areas of spiked internal standards. Internal standards used for quantitation (Cambridge Isotope Laboratories, D3-acetate DLM-3126-25; D5-propionate DLM-1601-1; D7-butyrate DLM-7616-PK; D9-valerate DLM-572-5 DLM-572-5; D8-valine DLM-488-0.25; D6-succinate DLM-831-PK; D6-phenol DLM-370-PK). Relative abundance data were normalized to the average of D8-valine/D6-valerate or D7-proline/D6-phenol. ***Metabolite Identification:*** Authentic standards were purchased for all targeted compounds and prepared at 1 mg/ml in methanol. All metabolite identifications are level 1 according to the CAWG standard initiative [2].

**Assay 2 (UPLC-QTOF-MS): Bile acid targeted metabolomics (-)ESI-LC-QTOF (quantitative and normalized relative abundance)**

***Extraction:*** For bile acid analysis, 75 µl of supernatant was added to pre-labeled microcentrifuge tubes and dried down completely under a nitrogen stream at 30 l/min (top) 1 l/min (bottom) at 30 °C (Biotage SPE Dry 96 Dual; 3579M). Samples were reconstituted in 50:50 water:methanol (750 µl) and added to a thermomixer C (Eppendorf) to resuspend analytes at 4 °C, 1000 rpm for 15 min with an infinite hold at 4 °C. Samples were then centrifuged at 4 °C, 20,000 x g for 15 min to remove insoluble debris, and the supernatant (700 µl) was transferred to fresh, pre-labeled mass spectrometry autosampler vials (Microliter; 09-1200) for LCMS analysis. ***Chromatography:*** Samples were analyzed using a liquid chromatography system (Agilent 1290 Infinity II). Each sample (5 µl) was injected onto an XBridge BEH C18 Column (3.5 µm, 2.1 x 100 mm; Waters Corporation, PN) fitted with an XBridge BEH C18 guard (Waters Corporation, PN) at 45 °C. Elution started with 72% A (Water, 0.1% formic acid) and 28% B (Acetone, 0.1% formic acid) with a flow rate of 0.4 ml/min for 1 min and linearly increased to 33% B over 5 min, and then linearly increased to 65% B over 14 min. The flow rate was then increased to 0.6 ml/min and B was increased to 98% over 0.5 min, and these conditions were held constant for 3.5 min. Finally, re-equilibration at a flow rate of 0.4 ml/min of 28% B was performed for 3 min. ***Instrument Settings:*** Samples were analyzed using a quadrupole time-of-flight (QTOF) mass spectrometer (Agilent 6546) (coupled with Agilent 1290 Infinity II) in negative mode, equipped with an Agilent Jet Stream Electrospray Ionization source. The electrospray ionization conditions were set with the capillary voltage at 3.5 kV, nozzle voltage at 2 kV, gas temperature 325 °C, gas flow 10 l/min, nebulizer 20 psig, sheath gas temperature 275 °C, and sheath gas flow 11 l/min. Data was acquired at a scan rate of 2 spectra/s, MS1 mode, and detection window set to 100-1700 m/z with continuous infusion of a reference mass (Agilent ESI TOF Biopolymer Analysis Reference Mix) for mass calibration. ***Data Transformation:*** A 10-point calibration curve was used for quantitation of 8 bile acids (cholic acid, glycocholic acid, taurocholic acid, deoxycholic acid, lithocholic acid, isodeoxycholic acid, alloisolithocholic acid and 3-oxolithocholic acid). Bile acid data analysis was performed using **MassHunter Profinder Analysis software (version B.10, Agilent Technologies)**. Identification of endogenous compounds was confirmed by comparison of m/z and retention time to authentic standards (**Supplementary Table 3**). Normalized peak areas were calculated by dividing raw peak areas of targeted analytes by averaged raw peak areas of spiked internal standards. ***Metabolite Identification:*** Authentic bile acid standards were prepared at 1 mg/ml in methanol and analyzed as pure standards as well as spiked into a pooled biological sample from each batch. **(Avanti Polar Lipids and Sigma Aldrich).** All metabolite identifications are level 1 according to the CAWG standard initiative [2]. Additionally, ions with epimer/isomers were purchased and analyzed individually and as mixtures to ensure accurate identification in biological samples. Compounds that were not resolvable with clear overlapping epimer/isomers were removed from analyses (if applicable). All identified compounds are reported at < 5 ppm difference between the known m/z and the detected m/z with a mass resolution of 50,000.

**Assay 3 (UPLC-TQ-MS): Desaminotyrosine (DAT) and indole-3-carboxaldehyde (I3C) targeted quantitation by (-)ESI-LC-QQQ (quantitative)**

***Extraction:*** For analysis of desaminotyrosine and indole-3-carboxyaldehyde, 200 µl of supernatant was added to pre-labeled microcentrifuge tubes and dried down completely under a nitrogen stream at 30 l/min (top) 1 l/min (bottom) at 30 °C (Biotage SPE Dry 96 Dual; 3579M). Samples were reconstituted in 50:50 water:methanol (50 µl) and added to a thermomixer C (Eppendorf) to resuspend analytes at 4 °C, 1000 rpm for 15 min with an infinite hold at 4 °C. Samples were then centrifuged at 4 °C, 20,000 x g for 15 min to remove insoluble debris, and supernatant (40 µl) from each sample was transferred to a 96 well plate (Agilent; 5065-4402) for LCMS analysis. ***Chromatography:*** Samples were analyzed on an Agilent 1290 infinity II liquid chromatography system. Each sample (2 µl) was injected into an Acquity UPLC HSS PFP column, 1.8 µm, 2.1 x 100 mm (Waters; 186005967) equipped with an Acquity UPLC HSS PFP VanGuard Pre-column, 100 Å, 1.8 µm, 2.1 mm x 5 mm (Waters; 186005974) at 45 °C. Mobile phase

A was 0.1% formic acid in water and mobile phase B was 0.1% formic acid in 95:5 acetonitrile:water. The flow rate was set to 0.5 ml/min starting at 0% B held constant for 3 min, then linearly increased to 50% over 5 min, then linearly increased to 95% B over 1 min, and held at 100% B for the next 3 min. Mobile phase B was then brought back down to 0% over 0.5 min and held at 0% for re-equilibration for 2.5 min. ***Instrument Settings:*** Samples were analyzed using an Agilent 6470 triple quadrupole mass spectrometer (coupled with Agilent 1290 Infinity II) in negative mode, equipped with an Agilent Jet Stream Electrospray Ionization source. The electrospray conditions were set with capillary voltage at 4 kV and nozzle voltage at 500 V, and Dynamic MRM was used with a cycle time of 500 ms. Sheath gas flow and temperature were 11 l/min and 275 °C, respectively. Transitions were monitored in negative mode, and the transitions for desaminotyrosine and indole-3-carboxaldehyde were 165.17 m/z to m/z 93.1 and 144.16 m/z to 116 m/z, respectively. DAT acquisition: Fragmentor (70), Collision Energy (32), Cell Accelerator Voltage (5). I3C acquisition: Fragmentor (90), Collision Energy (16), Cell Accelerator Voltage (5). ***Data Transformation:*** An 11-point calibration curve (ranging from 0.98 nM to 1 mM) was prepared using desaminotyrosine and indole-3-carboxaldehyde standards. Data analysis was performed using **MassHunter Quant software (version B.10, Agilent Technologies)** and confirmed by comparison of retention time, nominal mass, and qualifying fragment ion to authentic standards. Normalized peak areas were calculated by dividing raw peak areas of targeted analytes by averaged raw peak areas of spiked internal standards. ***Metabolite Identification:*** Stock solutions of desaminotyrosine and indole-3-carboxaldehyde (1 mg/ml in 50:50 water:methanol) were prepared using standards purchased from **Sigma Aldrich.** All metabolite identifications are level 1 according to the CAWG standard initiative [2].

References

1.Haak BW, Littmann ER, Chaubard JL, Pickard AJ, Fontana E, Adhi F, Gyaltshen Y, Ling L, Morjaria SM, Peled JU, van den Brink MR, Geyer AI, Cross JR, Pamer EG, Taur Y. Impact of gut colonization with butyrate-producing microbiota on respiratory viral infection following allo-HCT. *Blood.* 2018 Jun 28; 131(26):2978-2986. doi: 10.1182/blood-2018-01-828996. Epub 2018 Apr 19. PMID: 29674425; PMCID: PMC602463

2. Sumner LW, Amberg A, Barrett D, Beale MH, Beger R, Daykin CA, Fan TW, Fiehn O, Goodacre R, Griffin JL, Hankemeier T, Hardy N, Harnly J, Higashi R, Kopka J, Lane AN, Lindon JC, Marriott P, Nicholls AW, Reily MD, Thaden JJ, Viant MR. Proposed minimum reporting standards for chemical analysis Chemical Analysis Working Group (CAWG) Metabolomics Standards Initiative (MSI). Metabolomics. 2007 Sep;3(3):211-221. doi: 10.1007/s11306-007-0082-2. PMID: 24039616; PMCID: PMC3772505.
