# Supplementary material for: Immunomodulatory fecal metabolites are associated with mortality in COVID-19 patients with respiratory failure
Source: Nat Commun. 2022 Nov 3;13:6615. doi: 10.1038/s41467-022-34260-2 (PMC9633022; doi:10.1038/s41467-022-34260-2)
Supplement: Supplementary file 3 — Reporting Summary [file 41467_2022_34260_MOESM3_ESM.pdf]

nature portfolio

Corresponding author(s): Eric G. Pamer

Last updated by author(s): 2022-October 13

Reporting Summary

Nature Portfolio wishes to improve the reproducibility of the work that we publish. This form provides structure for consistency and transparency in reporting. For further information on Nature Portfolio policies, see our Editorial Policies and the Editorial Policy Checklist.

Statistics

For all statistical analyses, confirm that the following items are present in the figure legend, table legend, main text, or Methods section.

n/a	Confirmed	
☐	☒	The exact sample size (*n*) for each experimental group/condition, given as a discrete number and unit of measurement
☐	☒	A statement on whether measurements were taken from distinct samples or whether the same sample was measured repeatedly
☐	☒	The statistical test(s) used AND whether they are one- or two-sided<br>*Only common tests should be described solely by name; describe more complex techniques in the Methods section.*
☐	☒	A description of all covariates tested
☐	☒	A description of any assumptions or corrections, such as tests of normality and adjustment for multiple comparisons
☐	☒	A full description of the statistical parameters including central tendency (e.g. means) or other basic estimates (e.g. regression coefficient) AND variation (e.g. standard deviation) or associated estimates of uncertainty (e.g. confidence intervals)
☐	☒	For null hypothesis testing, the test statistic (e.g. *F*, *t*, *r*) with confidence intervals, effect sizes, degrees of freedom and *P* value noted<br>*Give P values as exact values whenever suitable.*
☒	☐	For Bayesian analysis, information on the choice of priors and Markov chain Monte Carlo settings
☒	☐	For hierarchical and complex designs, identification of the appropriate level for tests and full reporting of outcomes
☒	☐	Estimates of effect sizes (e.g. Cohen's *d*, Pearson's *r*), indicating how they were calculated

Our web collection on statistics for biologists contains articles on many of the points above.

Software and code

Policy information about availability of computer code

Data collection	All data collection were performed using R version 4.1.1 for large data pools. Clinical data were stored in REDcap version LTS 11.1.7
Data analysis	All analyses were performed using R version 4.1.1. All code used in this study for analyses and to generate figures is available both at GitHub (https://github.com/DFI-Bioinformatics/SARS-CoV-2) and Zenodo (https://doi.org/10.5281/zenodo.6858446).

For manuscripts utilizing custom algorithms or software that are central to the research but not yet described in published literature, software must be made available to editors and reviewers. We strongly encourage code deposition in a community repository (e.g. GitHub). See the Nature Portfolio guidelines for submitting code & software for further information.

Data

Policy information about availability of data

All manuscripts must include a data availability statement. This statement should provide the following information, where applicable:

- Accession codes, unique identifiers, or web links for publicly available datasets
- A description of any restrictions on data availability
- For clinical datasets or third party data, please ensure that the statement adheres to our policy

All data are available to the public. Raw sequencing data generated in this study have been deposited onto the National Center for Biotechnology Information (NCBI) Sequence Read Archive (SRA) under accession number PRJNA842425 [https://www.ncbi.nlm.nih.gov/bioproject/PRJNA842425]. Raw metabolomic data generated in this study have been deposited onto Metabolights under accession number MTBLS5288 [https://www.ebi.ac.uk/metabolights/MTBLS5288]. All processed data in this study are hosted on both GitHub (https://github.com/DFI-Bioinformatics/SARS-CoV-2) as well as Zenodo (https://doi.org/10.5281/zenodo.6858446).

Human research participants

Policy information about studies involving human research participants and Sex and Gender in Research.

Reporting on sex and gender	Self reported gender was collected by review of the electronic medical record. At the University of Chicago, patients are asked their gender at the time of admission and that data is recorded in the electronic medical record.
Population characteristics	The median age of the survival cohort was 58 and the deceased cohort was 66. Hyperlipidemia was present in 59% of survivors and 71% of those who passed away. Fifteen percent of survivors had a diagnosis of cancer and 9.4 of patients who passed away had a diagnosis of cancer.
Recruitment	Patients were recruited upon admission to the ICU and receipt of non-invasive mechanical ventilation, high flow nasal cannula, invasive mechanical ventilation or receipt of vasoactives. Our study did utilize surrogate consent in many cases. Patient selection bias was minimized as all patients with entry criteria were approached for consent 7 days a week in the ICU. If patients were not able to consent for themselves, a surrogate was approached to obtain consent.
Ethics oversight	This study was approved by the University of Chicago Institutional Review Board (IRB 20-1102) and has been registered at clinicaltrials.gov as NCT #04552834.

Note that full information on the approval of the study protocol must also be provided in the manuscript.

Field-specific reporting

Please select the one below that is the best fit for your research. If you are not sure, read the appropriate sections before making your selection.

☒ Life sciences ☐ Behavioural & social sciences ☐ Ecological, evolutionary & environmental sciences

For a reference copy of the document with all sections, see nature.com/documents/nr-reporting-summary-flat.pdf

Life sciences study design

All studies must disclose on these points even when the disclosure is negative.

Sample size	This study was a convenience sample enrolled during the COVID Pandemic from September 1 2020- May 2021. The study size was limited by the number of patients hospitalized for severe COVID-19 and yielded statistically significant associations between fecal metabolites and clinical ourtcomes. Previous microbiome observational studies in critically ill patients had sample sizes that ranged from 8-115 (Lankelma, J.M., et al., Intensive Care Med, 2017. 43(1): p. 59-68. McDonald, D., et al., mSphere, 2016. 1(4). Zaborin, A., et al. mBio, 2014. 5(5): p. e01361-14. Ojima, M., et al., Dig Dis Sci, 2016. 61(6): p. 1628-34.) Given that the fecal collection protocol was intended to assess the impact of microbiome on longitudinal long-term outcomes, the sample size calculation would be complex and would rely on simulation studies of pilot data (ferdous et al. Mucosal Immunol. 2022 Jul 22. doi: 10.1038/s41385-022-00548-1.), which does not yet exist. Therefore, we sought to obtain pilot data with this protocol to inform future sample size calculations and ensured the number of enrolled subjects was within the range of previously reported sample sizes.
Data exclusions	No data were excluded from the study.
Replication	Metagenomic sequencing and metabolomic assays have been replicated on a subset of samples and are highly reproducible. The clinical population and the fecal samples we have collected are unique and have not been replicated by investigation of a similar population.
Randomization	This was an observational cohort study and did not use randomization.
Blinding	This was an observational cohort study and did not require blinding

Reporting for specific materials, systems and methods

We require information from authors about some types of materials, experimental systems and methods used in many studies. Here, indicate whether each material, system or method listed is relevant to your study. If you are not sure if a list item applies to your research, read the appropriate section before selecting a response.

Materials & experimental systems

n/a	Involved in the study
☒	☐ Antibodies
☒	☐ Eukaryotic cell lines
☒	☐ Palaeontology and archaeology
☒	☐ Animals and other organisms
☐	☒ Clinical data
☒	☐ Dual use research of concern

Methods

n/a	Involved in the study
☒	☐ ChIP-seq
☒	☐ Flow cytometry
☒	☐ MRI-based neuroimaging

Clinical data

Policy information about clinical studies

All manuscripts should comply with the ICMJEguidelines for publication of clinical research and a completedCONSORT checklist must be included with all submissions.

Clinical trial registration: This study has been registered at clinicaltrials.gov as NCT #04552834.

Study protocol: the study protocol can be found at clinicaltrials.gov.

Data collection: Patients were enrolled at University of Chicago from September 2020 through May 2021 and data were collected on patients for one year following discharge from the MICU.

Outcomes: The clinical outcomes were pre-specified in our original protocol and included mortality (primary outcome) and ventilator days (i.e. need for mechanical ventilation; secondary outcome). These outcomes were prospectively collected based on clinical documentation during the hospitalization and survival after discharge was confirmed with health records, phone calls, and social security death index.
